# Exploring the Relationship Between Participation in Leisure Sports Activities, Health Behaviors, and Life Satisfaction Among Older Adults with Disabilities

**DOI:** 10.3390/medicina61040713

**Published:** 2025-04-12

**Authors:** Seungok An, Su-Yeon Roh, Jeonga Kwon

**Affiliations:** 1 Department of Special Physical Education, Yongin University, Yongin 17092, Republic of Korea; skyforve@yiu.ac.kr; 2 Department of Exercise Rehabilitation, Gachon University, Incheon 21936, Republic of Korea; 3 Department of Elementary Education, College of First, Korea National University of Education, Cheongju 28173, Republic of Korea

**Keywords:** disabilities, older adults, health behavior, leisure sports activities, life satisfaction

## Abstract

*Background and Objectives*: As the world enters an aging society, it is necessary to focus on older individuals, especially those with disabilities. The latter must face two issues, their disability and their age; therefore, a lot of attention must be paid to their health. In this study, we aimed to explore the association between participation in leisure sports activities, health behaviors, and life satisfaction among older adults with disabilities. The research question we aimed to address was as follows: Can participation in leisure sports activities positively affect the health behaviors and life satisfaction of older adults with disabilities? *Materials and Methods*: Data were sourced from the 2023 Survey on the Status of Persons with Disabilities organized by the Korea Ministry of Health and Welfare. The collected data were analyzed using frequency analyses, chi-squared tests, and multivariate logistic regression analyses. *Results*: Multivariate logistic regression analyses revealed that regarding sex, the average odds ratio (OR) was 1.525 (*p* = 0.001) for males. Regarding efforts to control weight, the average OR was 1.808 (*p* < 0.001) for “tried to reduce”, 1.893 (*p* < 0.001) for “tried to maintain”, and 1.245 (*p* = 0.345) for “tried to increase”. Regarding smoking, the average OR was 0.612 (*p* = 0.008) for “smoke daily”, 0.947 (*p* = 0.889) for “smoke occasionally”, and 1.175 (*p* = 0.255) for “smoked in the past but do not smoke now”. Regarding health status satisfaction, the average OR was 2.014 (*p* = 0.022) for very satisfied, 1.846 (*p* = 0.013) for somewhat satisfied, and 1.347 (*p* = 0.220) for somewhat dissatisfied. Regarding friendship satisfaction, the average OR was 9.177 (*p* < 0.001) for very satisfied, 5.428 (*p* < 0.001) for somewhat satisfied, and 3.024 (*p* = 0.011) for somewhat dissatisfied. Namely, participation in leisure sports activities was significantly related to weight control efforts, smoking cessation, increased health status satisfaction, and increased friendship satisfaction among older adults with disabilities. *Conclusions*: Access to leisure sports activities should be increased to improve the health of older adults with disabilities. Furthermore, leisure sports activities should be considered in the establishment of policies and services to enhance the health of these individuals.

## 1. Introduction

Many countries worldwide are encountering issues related to an aging population, and Korea is on the verge of becoming a super-aging society. As the proportion of older adults in the population increases, various problems related to health, poverty, and loneliness are prevalent. Older adults experience degeneration in several forms, such as physical, biological, biochemical, and psychological. However, older adults with disabilities experience difficulties not only related to aging but also their disability, and among the various related problems, health is receiving more attention. Older individuals with disabilities face challenges concerning health and independent living. Therefore, the degree of difficulty experienced in this group is greater than that of older people without disabilities and young people with disabilities [1,2]. Approximately 87% of adults with disabilities have secondary conditions, and they are more likely to have secondary diseases than adults without disabilities [1]. Older adults with disabilities may experience discrimination due to false prejudice about their disability and age [3]. This discrimination can lead to poverty due to low employment and wages, poor relationships, and loneliness because of social alienation. It can also reduce life satisfaction and hamper their well-being [4,5].

Individuals with disabilities generally have lower life satisfaction than those without disabilities because their daily activities are limited [6,7]. A study conducted across 132 countries found that health and lifestyle satisfaction decreases among older adults with increasing age and disability levels [8]. However, studies have reported that participating in leisure sports activities positively affects the quality of life and subjective well-being of older adults with disabilities [9,10]. This finding suggests that the participation of older individuals with disabilities in leisure sports activities revitalizes their lives and positively affects their attitudes and perspectives.

Individuals with disabilities are less likely to practice positive health behaviors, such as quitting smoking, abstaining from alcohol, and eating healthy foods, than those without disabilities [11,12,13]. Kim et al. [12] reported that older adults with disabilities have more undesirable lifestyle risk factors than those without disabilities. Courtney-Long et al. [13] found that adults with disabilities are more likely to be current smokers than adults without disabilities. Moreover, as disability increases, unhealthy diet, physical inactivity, and obesity become more prevalent [12]. These negative health behaviors can exacerbate the severity of pre-existing disabilities. In fact, the risk of disability increases with a decrease in the number of positive health behaviors [11]. Older adults with disabilities, in particular, are more likely to have secondary diseases than those without disabilities [1]. Practicing positive health behaviors can reduce the likelihood of secondary diseases among these individuals [12].

Older adults with disabilities can live a healthy life in many ways; participating in leisure sports activities is one of them. Older adults with disabilities are more likely to have a cascading chain of illnesses [1]. Physical inactivity exacerbates their secondary illnesses and increases the risk of chronic diseases, which leads to more disabilities [14]. Participation in leisure sports activities enhances the quality of life of older adults with disabilities, reduces the risk of mortality [15,16], and bolsters functional abilities, thus helping them maintain independence in performing activities of daily living [17]. In other words, participation in leisure sports activities can help older adults with disabilities prevent secondary illnesses and live healthy lives.

Other studies have separately examined participation in leisure sports activities, practice of health behaviors, and life satisfaction among older adults with disabilities. However, no study has examined how participation in leisure sports activities is associated with different components of health behaviors and life satisfaction [14,15,16,17]. Thus, it can be inferred that few studies have comprehensively examined the health of older adults with disabilities. In Republic of Korea, there is a widespread perception that “individuals with disabilities are unhealthy”; consequently, the health issues faced by these individuals remain relatively neglected [18,19]. This makes it imperative to determine the current status of participation in leisure sports activities, practice of health behaviors, and life satisfaction among older adults with disabilities. It is also vital to explore how their participation in leisure sports activities is related to different aspects of health behaviors and life satisfaction.

In Republic of Korea, older adults with disabilities experience disability and aging, as well as negative social perceptions, and their health issues have remained unresolved. Exploring their participation in leisure sports activities, practice of health behaviors, and life satisfaction can help improve their lives. The results of this study will provide foundational data for understanding the health of this demographic. Moreover, the findings can be used to identify ways through which the lives of older adults with disabilities can be enhanced. Therefore, in this study, we explored how participation in leisure sports activities is associated with health behaviors and life satisfaction among Korean older adults with disabilities. The research question we aimed to address was as follows: Can participation in leisure sports activities positively affect the health behaviors and life satisfaction of older adults with disabilities?

## 2. Materials and Methods

### 2.1. Design and Study Population

This study was conducted using data from the 2023 Survey on the Status of Persons with Disabilities organized by the Korea Ministry of Health and Welfare. The survey included 8000 Korean individuals with disabilities recruited using two-stage cluster sampling. A sample was extracted from 8000 individuals registered as having disabilities. These individuals were those living in general residential facilities in 17 cities and provinces across the country while considering their degree of disability. A researcher visited the participants’ homes and conducted in-depth interviews using a structured questionnaire. The Korea Ministry of Health and Welfare deletes personally identifiable information, such as names, phone numbers, and email addresses, from the survey data. After completing a data access form and data use pledge (security memorandum), as well as submitting the research plan, we obtained the secondary data with the approval of the Korea Ministry of Health and Welfare (https://data.kihasa.re.kr/kihasa/kor/contents/ContentsList.html, accessed on 9 March 2025). From the survey, we used the data of 4919 respondents aged 60 or older. This study was approved by the Institutional Review Board of the Korea Ministry of Health and Welfare (approval number 117032; 31 December 2022). All participants were informed of the study’s purpose, and they voluntarily signed an informed consent form. Furthermore, this study was conducted according to the principles outlined in the Declaration of Helsinki.

### 2.2. Independent Variable

The independent variable was participation in leisure sports activities. Leisure sports are those in an intermediate stage between a simple relaxing walk and the traditional definition of sport. Leisure sports have some sports characteristics, but they do not pursue achievement through matches and do not require regular, intense training. Instead, the goal is to seek physical relaxation and comfort through informal and voluntary activities [20]. We used this definition of leisure sports for the independent variable question. The respondents were asked, “In the past week, did you play any sport (for example, football, tennis, or swimming) as a leisure activity?” The response options were yes and no. We used the responses without any modifications.

### 2.3. Dependent Variables

The dependent variables were sex, age, health behaviors, and life satisfaction. Each variable was categorized into the variables of health behaviors and life satisfaction, considering their characteristics. For example, health behaviors comprised eating regular meals, having a balanced diet, making efforts to control weight, stress in daily life, measuring body composition, smoking, and drinking. The components of life satisfaction were health status satisfaction, life satisfaction, and friendship satisfaction.

Whether one ate regular meals was determined by asking, “How consistent are you with eating your meals on time?” The response options were “regular”, “sometimes irregular”, and “irregular”. Whether one ate a balanced diet was identified by asking, “Do you have nutritionally balanced meals?” The response options were “yes”, “sometimes”, and “no”. Whether one made efforts to control one’s weight was asked using this question: “Have you ever tried to control your weight for an entire year?” The response options were “tried to reduce”, “tried to maintain”, “tried to increase”, and “never tried to control”. The degree of perceived stress was determined using this question: “How stressed do you feel in daily life?” The response options were “extremely stressed”, “moderately stressed”, “slightly stressed”, and “not at all stressed”. Whether one measured their body composition was assessed by asking respondents, “Have you measured your height or weight recently?” The response options were “yes”, “no”, and “cannot be measured”. In this study, “cannot be measured” was considered to be “no”, and the responses were subsequently categorized as “yes” or “no”. Whether one smoked was determined using this question: “Do you smoke?” The response options were “smoke daily”, “smoke occasionally”, “smoked in the past but do not smoke now”, and “never smoked and do not currently smoke”. Whether one consumed alcohol was asked using this question: “How often do you drink?” The response options were “do not drink”, “less than once a month”, “about once a month”, “2–4 times a month”, “about 2–3 times a week”, and “more than 4 times a week”. In this study, “do not drink”, “less than once a month”, and “about once a month” were grouped as “less than once a month”. Therefore, the responses were categorized as “less than once a month”, “2–4 times a month”, “about 2–3 times a week”, and “more than 4 times a week”.

Health status satisfaction was assessed by asking respondents, “How satisfied are you with your current state of health?” The response options were “very satisfied”, “somewhat satisfied”, “somewhat dissatisfied”, and “very dissatisfied”. Life satisfaction was determined using this question: “How satisfied are you with your current life?” The response options were “very satisfied”, “somewhat satisfied”, “somewhat dissatisfied”, and “very dissatisfied”. Friendship satisfaction was evaluated using this question: “How satisfied are you with the number of friends you have and your relationships?” The response options were “very satisfied”, “somewhat satisfied”, “somewhat dissatisfied”, and “very dissatisfied”.

### 2.4. Statistical Analysis

The collected data were analyzed in the following manner. First, a frequency analysis was conducted on the characteristics of the study population, including their participation in leisure sports activities, health behaviors, and life satisfaction. Second, we conducted chi-squared tests to identify differences in the characteristics of the study population based on their participation in leisure sports activities. Third, we performed multivariate logistic regression analyses to analyze how participation in leisure sports activities was associated with health behaviors and life satisfaction. Multivariate logistic regression analysis is a statistical technique that helps to determine the relationship between the independent and dependent variables. Because the purpose of this study was to investigate the association between participation in leisure sports activities, health behaviors, and life satisfaction among older adults with disabilities, this technique was considered suitable for addressing the research question. Odds ratios (ORs), 95% confidence intervals (CIs), and *p*-values are presented. All statistical analyses were performed using SPSS version 23.0 for Windows (IBM Corp., Armonk, NY, USA), and the statistical significance was set at *p* < 0.05. The researchers participated in statistics-related training. In addition, peer verification was conducted with external researchers who had participated in various studies using multivariate logistic regression analyses to confirm the reliability and validity of data collection and statistical processing.

## 3. Results

### 3.1. Participant Characteristics

As shown in Table 1, among the 4919 respondents, most were male (56.9%). Regarding participation in leisure sports activities, most respondents did not participate (96.2%). Regarding health behaviors, most respondents had regular meals (71.6%) and ate a balanced diet either sometimes (47.3%) or always (41.8%). The majority of respondents had never tried to control their weight (61.4%). Many respondents reported feeling a little stressed in daily life (44.0%). Most respondents measured their body composition (95.4%). The majority of the respondents consumed alcohol less than once a month (84.8%) and had never smoked and did not smoke at the time (55.4%). Regarding life satisfaction, a significant number of individuals were somewhat dissatisfied with their health status (44.5%). Most respondents were somewhat satisfied with their lives (54.9%), and many reported being somewhat satisfied with their friendships (48.1%).

### 3.2. Chi-Squared Tests

Table 2 presents the results of the chi-squared tests. Sex (χ2 = 6.594, *p* = 0.011), age (χ2 = 21.899, *p* < 0.001), balanced diet (χ2 = 31.850, *p* < 0.001), efforts to control weight (χ2 = 56.914, *p* < 0.001), stress (χ2 = 14.516, *p* = 0.002), body composition measurement (χ2 = 7.317, *p* = 0.002), smoking (χ2 = 8.055, *p* = 0.045), drinking (χ2 = 29.125, *p* < 0.001), health status satisfaction (χ2 = 55.529, *p* < 0.001), life satisfaction (χ2 = 73.389, *p* < 0.001), and friendship satisfaction (χ2 = 109.471, *p* < 0.001) differed significantly based on participation in leisure sports activities.

### 3.3. Multivariate Logistic Regression Analyses

Table 3 shows the results of the multivariate logistic regression analyses. Regarding sex, the average OR was 1.525 (95% CI, 1.188–1.958, *p* = 0.001) for males. Regarding regular meals, the average OR was 0.985 (95% CI, 0.587–1.655, *p* = 0.956) for regular and 1.002 (95% CI, 0.597–1.683, *p* = 0.993) for sometimes irregular. Regarding balanced diet, the average OR was 1.576 (95% CI, 0.993–2.502, *p* = 0.054) for yes and 1.120 (95% CI, 0.714–1.757, *p* = 0.621) for sometimes. Regarding efforts to control weight, the average OR was 1.808 (95% CI, 1.428–2.289, *p* < 0.001) for tried to reduce, 1.893 (95% CI, 1.464–2.449, *p* < 0.001) for tried to maintain, and 1.245 (95% CI, 0.790–1.961, *p* = 0.345) for tried to increase. Regarding stress, the average OR was 1.434 (95% CI, 0.851–2.417, *p* = 0.176) for feeling every moment, 0.908 (95% CI, 0.664–1.242, *p* = 0.546) for feeling a lot, and 1.155 (95% CI, 0.906–1.472, *p* = 0.244) for feeling a little. Regarding body composition measurement, the average OR was 1.715 (95% CI, 0.749–3.927, *p* = 0.202) for yes.

Regarding smoking, the average OR was 0.612 (95% CI, 0.427–0.877, *p* = 0.008) for smoke daily, 0.947 (95% CI, 0.439–2.041, *p* = 0.889) for smoke occasionally, and 1.175 (95% CI, 0.890–1.551, *p* = 0.255) for smoked in the past but do not smoke now. Regarding drinking, the average OR was 0.896 (95% CI, 0.507–1.585, *p* = 0.707) for less than once a month, 1.143 (95% CI, 0.623–2.097, *p* = 0.667) for two to four times a month, and 1.091 (95% CI, 0.579–2.055, *p* = 0.787) for about two to three times a week. Regarding health status satisfaction, the average OR was 2.014 (95% CI, 1.108–3.663, *p* = 0.022) for very satisfied, 1.846 (95% CI, 1.138–2.993, *p* = 0.013) for somewhat satisfied, and 1.347 (95% CI, 0.837–2.168, *p* = 0.220) for somewhat dissatisfied. Regarding life satisfaction, the average OR was 1.336 (95% CI, 0.590–3.026, *p* = 0.487) for very satisfied, 1.220 (95% CI, 0.568–2.621, *p* = 0.610) for somewhat satisfied, and 0.632 (95% CI, 0.295–1.353, *p* = 0.237) for somewhat dissatisfied. Regarding friendship satisfaction, the average OR was 9.177 (95% CI, 3.894–21.628, *p* < 0.001) for very satisfied, 5.428 (95% CI, 2.348–12.548, *p* < 0.001) for somewhat satisfied, and 3.024 (95% CI, 1.287–7.102, *p* = 0.011) for somewhat dissatisfied.

## 4. Discussion

This study yielded several insightful results. First, older males with disabilities were more likely to participate in leisure sports activities than older females with disabilities. Greater participation among males may result from increased health concerns with age and more available time owing to retirement [21,22]. Conversely, lesser participation among females may stem from sex-based differences regarding barriers to participation in sports activities. Although males and females can participate in sports in modern society, it is still considered exclusive to males as it symbolizes masculinity. For example, females who are good at sports are often misunderstood, and societal prejudice exists where they are sometimes considered masculine. This confirms that sports participation is viewed differently according to sex [21,22,23]. In particular, older females with disabilities face three types of barriers: those associated with their sex, disability, and age. These barriers may make it challenging for older females with disabilities to participate in leisure sports activities [23]. Because of these social and cultural factors, older females with disabilities are less likely to participate in leisure sports activities than older males with disabilities.

Second, participation in leisure sports activities was significantly related to weight control efforts among older adults with disabilities. This finding suggests that participation in leisure sports activities positively affects the voluntary weight control practices of older adults with disabilities. Individuals with disabilities are less likely to engage in physical activity and have difficulty maintaining an appropriate weight because of limited access to health-promoting environments, such as gyms and parks [24]. These limitations put them at a greater risk of becoming obese than individuals without disabilities [25]. However, the risk varies based on the type of disability. For people with upper limb impairments, their restricted physical activity also limits their dietary activity, which mitigates the risk of obesity [26]. In contrast, people with lower limb impairments or spinal cord injuries are more susceptible to obesity owing to their greatly limited physical activity and low energy metabolism, coupled with their relatively preserved bodily functions for eating [26]. Hossaini et al. [24] argued that physical activity and diet affect the weight control of individuals with disabilities, and physical activity- and diet-related interventions can help them lose weight. Overall, older adults with disabilities can use physical activity to control their weight, and physical activity may be beneficial in mitigating the obesity issue among these individuals. However, additional physical activity programs may be required for those with upper limb impairments.

Third, participation in leisure sports activities was significantly related to smoking cessation among older adults with disabilities. This finding suggests that participation in leisure sports activities prompts older adults with disabilities to quit smoking. It aligns with the findings of previous studies showing a significant association between participation in leisure sports and smoking cessation among older adults without disabilities [27], adults with disabilities [28], and adults without disabilities [29]. Participation in leisure sports activities positively affects smoking cessation, regardless of who participates [27,28,29]. Notably, the prevalence of smoking is more than 10% higher among individuals with disabilities than the prevalence among the general population. Additionally, individuals with disabilities face disproportionate tobacco-related health disparities and poor access to effective treatment [30]. Studies have suggested that they tend to mitigate their psychological burden or dissatisfaction with their disability through smoking [31]. In particular, team-based leisure sports are effective in smoking cessation; therefore, older adults with disabilities can participate in team-based leisure sports to further enhance the effectiveness of smoking cessation [32]. This suggests that these individuals can reduce smoking behavior by participating in leisure sports with multiple people, which may resolve negative emotions related to disability through physical activity and emotional exchange.

Fourth, participation in leisure sports activities was significantly related to increased health status satisfaction among older adults with disabilities. Among individuals with disabilities, factors that influence perceived health status include disability, the severity of health status, and age [33,34]. People with disabilities report poorer physical and mental health than those without disabilities [35]. However, it is important to note that despite having a severe disability, an individual’s perceptions of and satisfaction with their health status may be high despite disparities with their objective health status [36]. This concept refers to the “disability paradox”, where people with the same disability can experience varying degrees and types of limitations in their activities based on environmental and personal factors [36]. Despite having a serious disability or disease, people with disabilities may experience a higher quality of life and have higher life satisfaction than that observed in non-disabled people [36]. This is possible through social support and acceptance of disabilities. Through continuous efforts and changes, people with disabilities can improve their quality of life compared with that of non-disabled people [37]. Our results support the disability paradox and suggest that the participation of older adults with disabilities in leisure sports activities may be a determinant of the paradox. Our results also indicate that participation in leisure sports activities helps older adults with disabilities live with a healthy body and mind.

Fifth, participation in leisure sports activities was significantly related to increased satisfaction with friendships among older adults with disabilities. This finding suggests that participation in leisure sports activities strengthens their relationships with others and is beneficial for social health. Participating in recreational sports improves social and communication skills as people cooperate with and care for others [38,39,40]. The benefits of participation in leisure sports activities also apply to older adults with disabilities and influenced our results. Notably, participation in sports activities has been reported to improve the social skills of individuals with autism spectrum disorder [39]. Studies have also demonstrated that participation in leisure sports activities reduces loneliness among individuals with disabilities who have severe limitations in their daily lives [40]. Companionship in shared leisure sports activity appears to provide effective relief for people as they experience daily life stress. In addition, many leisure sports activities can provide feelings of support [41]. As individuals age, they are more exposed to loneliness, and those with disabilities are even more socially isolated. In this respect, leisure sports play a decisive role in mediating loneliness as they can motivate older adults with disabilities to voluntarily participate in these activities [42]. Taken together, it can be inferred that participation in leisure sports activities can expand social interactions and increase friendship satisfaction among older adults with disabilities who experience loneliness and isolation. Moreover, it suggests the need for social support and financial support so that older individuals with disabilities can take care of their physical and mental health through leisure sports. In summary, participation in leisure sports activities is significantly related to weight control efforts, smoking cessation, and increased satisfaction with health status and friendships.

This study has several limitations. First, because this was a cross-sectional study, it was difficult to establish clear causal relationships between participation in leisure sports activities, health behaviors, and life satisfaction. Longitudinal or interventional studies are needed to accurately determine causality. Second, we used secondary data and did not consider scientific health data in examining the effects of participation in leisure sports activities on health behaviors and life satisfaction. Third, we did not categorize the type of disability. Various types of disabilities exist, such as visual impairment, hearing impairment, and developmental disabilities, but these were not categorized. Fourth, although this study was conducted among individuals who had a disability and were older, the results might have stemmed from the influence of one of these factors. Future studies must be designed with these limitations in mind to obtain specific and accurate findings. Fifth, the independent variable question we used to assess participation in leisure sports activities might not have reflected the types of activities that are realistically accessible or commonly practiced by older adults with disabilities. This could have led to the underreporting of participation as respondents might not have considered less intensive or non-competitive activities to fall under the definition of leisure sports. These limitations could have influenced our results. Nonetheless, this study is valuable because it used national data to investigate the relationship between participation in leisure sports activities, health behaviors, and life satisfaction. As such, we provide foundational data that can improve the health of older adults with disabilities and help them live healthy lives.

## 5. Conclusions

Participation in leisure sports activities is significantly related to weight control efforts, smoking cessation, and increased satisfaction with health status and friendships. Therefore, access to leisure sports activities should be increased to improve the health of older adults with disabilities. In modern society, supporting the health of older adults with disabilities is essential not only for their survival but also for them to lead happy lives. Participating in physical activity is low cost and highly effective. If older adults with disabilities participate in physical activity consistently, their health and quality of life will be enhanced. Notably, the number of older adults with disabilities is increasing, but policies to support their health are still insufficient. Leisure sports activities must be considered when formulating policies and services for these individuals. Such policies and services must aim to improve the health of older adults with disabilities and help them lead a happy life.

## Figures and Tables

**Table 1:** Characteristics of the study population (*n* = 4919).

Characteristic	Categories	*n* (%)
Participation in leisure sports activities	Yes	188 (3.8%)
	No	4731 (96.2%)
Sex	Male	2797 (56.9%)
	Female	2122 (43.1%)
Age	60–69	1929 (39.2%)
	70–79	1669 (33.9%)
	80 or older	1321 (26.9%)
Regular meals	Regular	3522 (71.6%)
	Sometimes irregular	1150 (23.4%)
	Irregular	247 (5.0%)
Balanced diet	Yes	2056 (41.8%)
	Sometimes	2327 (47.3%)
	No	536 (10.9%)
Efforts to control weight	Tried to reduce	861 (17.5%)
	Tried to maintain	757 (15.4%)
	Tried to increase	279 (5.7%)
	Never tried to control	3022 (61.4%)
Stress	Extremely stressed	270 (5.5%)
	Moderately stressed	1332 (27.1%)
	Slightly stressed	2166 (44.0%)
	Not at all stressed	1151 (23.4%)
Body composition measurement	Yes	4694 (95.4%)
	No	225 (4.6%)
Smoking	Smoke daily	472 (9.6%)
	Smoke occasionally	69 (1.4%)
	Smoked in the past but do not smoke now	1653 (33.6%)
	Never smoked and do not currently smoke	2725 (55.4%)
Drinking	Less than once a month	4172 (84.8%)
	2–4 times a month	310 (6.3%)
	About 2–3 times a week	250 (5.1%)
	More than 4 times a week	197 (3.8%)
Health status satisfaction	Very satisfied	97 (2.0%)
	Somewhat satisfied	1520 (30.9%)
	Somewhat dissatisfied	2190 (44.5%)
	Very dissatisfied	1112 (22.6%)
Life satisfaction	Very satisfied	276 (5.6%)
	Somewhat satisfied	2699 (54.9%)
	Somewhat dissatisfied	1592 (32.4%)
	Very dissatisfied	352 (7.2%)
Friendship satisfaction	Very satisfied	580 (11.8%)
	Somewhat satisfied	2367 (48.1%)
	Somewhat dissatisfied	1476 (30.0%)
	Very dissatisfied	496 (10.1%)

**Table 2:** Differences in the characteristics of the study population based on their participation in leisure sports activities.

Characteristic	Categories	Participation in Leisure Sports Activities	Non-Participation in Leisure Sports Activities	χ2 (*p*)
Sex	Male	124 (66.0%)	2673 (56.5%)	6.594 (0.011 *)
	Female	64 (34.0%)	2058 (43.5%)	
Age	60–69	93 (49.5%)	1836 (38.8%)	21.899 (<0.001 ***)
	70–79	72 (38.3%)	1597 (33.8%)	
	80 or older	23 (12.2%)	1298 (27.4%)	
Regular meals	Regular	142 (75.5%)	3380 (71.4%)	2.800 (0.247)
	Sometimes irregular	41 (21.8%)	1109 (23.4%)	
	Irregular	5 (2.7%)	242 (5.2%)	
Balanced diet	Yes	116 (61.7%)	1940 (41.0%)	31.850 (<0.001 ***)
	Sometimes	59 (31.4%)	2268 (47.9%)	
	No	13 (6.9%)	523 (11.1%)	
Efforts to control weight	Tried to reduce	62 (33.0%)	799 (16.9%)	56.914 (<0.001 ***)
	Tried to maintain	58 (30.8%)	699 (14.7%)	
	Tried to increase	10 (5.3%)	269 (5.7%)	
	Never tried to control	58 (30.9%)	2964 (62.7%)	
Stress	Extremely stressed	7 (3.7%)	263 (5.6%)	14.516 (0.002 **)
	Moderately stressed	31 (16.5%)	1301 (27.5%)	
	Slightly stressed	93 (49.5%)	2073 (43.8%)	
	Not at all stressed	57 (30.3%)	1094 (23.1%)	
Body composition measurement	Yes	187 (99.5%)	4507 (95.3%)	7.317 (0.002 **)
	No	1 (0.5%)	224 (4.7%)	
Smoking	Smoke daily	14 (7.4%)	458 (9.7%)	8.055 (0.045 *)
	Smoke occasionally	2 (1.1%)	67 (1.4%)	
	Smoked in the past but do not smoke now	81 (43.1%)	1572 (33.2%)	
	Never smoked and do not currently smoke	91 (48.4%)	2634 (55.7%)	
Drinking	Less than once a month	138 (73.4%)	4034 (85.3%)	29.125 (<0.001 ***)
	2–4 times a month	28 (14.9%)	282 (6.0%)	
	About 2–3 times a week	15 (8.0%)	235 (5.0%)	
	More than 4 times a week	7 (3.7%)	180 (3.8%)	
Health status satisfaction	Very satisfied	9 (4.8%)	88 (1.9%)	55.529 (<0.001 ***)
	Somewhat satisfied	95 (50.5%)	1425 (30.1%)	
	Somewhat dissatisfied	71 (37.8%)	2119 (44.8%)	
	Very dissatisfied	13 (6.9%)	1099 (23.2%)	
Life satisfaction	Very satisfied	25 (13.3%)	251 (5.3%)	73.389 (<0.001 ***)
	Somewhat satisfied	141 (75.0%)	2558 (54.1%)	
	Somewhat dissatisfied	19 (10.1%)	1573 (33.2%)	
	Very dissatisfied	3 (1.6%)	349 (7.4%)	
Friendship satisfaction	Very satisfied	58 (30.9%)	522 (11.0%)	109.471 (<0.001 ***)
	Somewhat satisfied	111 (59.0%)	2256 (47.8%)	
	Somewhat dissatisfied	17 (9.0%)	1459 (30.8%)	
	Very dissatisfied	2 (1.1%)	494 (10.4%)	

* *p* < 0.05, ** *p* < 0.01, *** *p* < 0.001; assessed through chi-squared tests.

**Table 3:** Association of participation in leisure sports activities with health behaviors and life satisfaction.

Characteristic	Categories	ORs	95% CIs	*p*-Value
Sex	Male	1.525	1.188–1.958	0.001 **
	Female	Reference		
Regular meals	Regular	0.985	0.587–1.655	0.956
	Sometimes irregular	1.002	0.597–1.683	0.993
	Irregular	Reference		
Balanced diet	Yes	1.576	0.993–2.502	0.054
	Sometimes	1.120	0.714–1.757	0.621
	No	Reference		
Efforts to control weight	Tried to reduce	1.808	1.428–2.289	<0.001 ***
	Tried to maintain	1.893	1.464–2.449	<0.001 ***
	Tried to increase	1.245	0.790–1.961	0.345
	Never tried to control	Reference		
Stress	Extremely stressed	1.434	0.851–2.417	0.176
	Moderately stressed	0.908	0.664–1.242	0.546
	Slightly stressed	1.155	0.906–1.472	0.244
	Not at all stressed	Reference		
Body composition measurement	Yes	1.715	0.749–3.927	0.202
	No	Reference		
Smoking	Smoke daily	0.612	0.427–0.877	0.008 **
	Smoke occasionally	0.947	0.439–2.041	0.889
	Smoked in the past but do not smoke now	1.175	0.890–1.551	0.255
	Never smoked and do not currently smoke	Reference		
Drinking	Less than once a month	0.896	0.507–1.585	0.707
	2–4 times a month	1.143	0.623–2.097	0.667
	About 2–3 times a week	1.091	0.579–2.055	0.787
	More than 4 times a week	Reference		
Health status satisfaction	Very satisfied	2.014	1.108–3.663	0.022 *
	Somewhat satisfied	1.846	1.138–2.993	0.013 *
	Somewhat dissatisfied	1.347	0.837–2.168	0.220
	Very dissatisfied	Reference		
Life satisfaction	Very satisfied	1.336	0.590–3.026	0.487
	Somewhat satisfied	1.220	0.568–2.621	0.610
	Somewhat dissatisfied	0.632	0.295–1.353	0.237
	Very dissatisfied	Reference		
Friendship satisfaction	Very satisfied	9.177	3.894–21.628	<0.001 ***
	Somewhat satisfied	5.428	2.348–12.548	<0.001 ***
	Somewhat dissatisfied	3.024	1.287–7.102	0.011 *
	Very dissatisfied	Reference		

* *p* < 0.05, ** *p* < 0.01, *** *p* < 0.001; assessed through multivariate logistic regression analysis. OR, odds ratio; CI, confidence interval.

## Data Availability

Publicly available datasets were analyzed in this study. These data can be found here: https://data.kihasa.re.kr/kihasa/kor/contents/ContentsList.html (accessed on 9 March 2025).
